# Plasma von Willebrand factor and endothelial-lineage flow-cytometry signals in acute pediatric IgA vasculitis: a cross-sectional pilot study

**DOI:** 10.3389/fped.2026.1930009

**Published:** 2026-09-03

**Authors:** Zichao Ding, Xianqing Ren, Hang Su, Min Tong, Xiaoqi Xu, Yingying Jiang

**Affiliations:** 1 Pediatric Hospital of the First Affiliated Hospital of Henan University of Chinese Medicine, Zhengzhou, China; 2 College of Pediatrics, Henan University of Chinese Medicine, Zhengzhou, China

**Keywords:** endothelial activation, flow cytometry, IgA vasculitis, pediatric rheumatology, pilot study, von Willebrand factor

## Abstract

**Introduction:**

Endothelial activation is implicated in childhood IgA vasculitis, but candidate endothelial markers may reflect systemic inflammation rather than endothelial perturbation.

**Methods:**

In this single-centre cross-sectional pilot study, we enrolled 13 children with acute IgA vasculitis and 20 age- and sex-comparable healthy controls screened to exclude acute infection, allergic disease and other inflammatory conditions. Plasma soluble fms-like tyrosine kinase 1, soluble vascular endothelial cadherin, von Willebrand factor (vWF) and vascular endothelial growth factor were measured by enzyme-linked immunosorbent assay (ELISA); CD45-low CD31^+^CD146^+^ endothelial-lineage events were quantified by three-colour flow cytometry in a subset.

**Results:**

Children with IgA vasculitis had higher C-reactive protein, neutrophil proportion and platelet count than controls. In the pooled analysis, vWF was higher in patients than controls (median 811.1 vs. 439.9 ng/mL; Hodges-Lehmann difference 335 ng/mL, 95% confidence interval 133-539; *P* = 0.008; Benjamini-Hochberg *q* = 0.03), but this contrast did not persist after ELISA plate stratification (van Elteren *P* = 0.38) and is therefore hypothesis-generating. Within patients, no clear monotonic associations were detected between vWF and C-reactive protein or neutrophil indices; these exploratory analyses were underpowered to establish independence from systemic inflammation. No clear group differences were detected in the other soluble analytes. Endothelial-lineage events were nominally higher in patients, but this readout was strongly affected by CD45-low gate size and acquisition-session variability.

**Discussion:**

These findings support batch-controlled validation of vWF and more rigorous endothelial-cell enumeration in future pediatric IgA vasculitis studies.

## Introduction

IgA vasculitis (IgAV), historically Henoch–Schönlein purpura, is the most common primary systemic vasculitis of childhood, with an annual incidence of approximately 10–27 per 100,000 children. It typically presents with non-thrombocytopenic palpable purpura, arthralgia or arthritis, gastrointestinal symptoms, and a variable risk of glomerulonephritis (1–3). The pathogenic backbone is deposition of galactose-deficient IgA1–containing immune complexes in small-vessel walls, with complement activation, neutrophil recruitment and release of neutrophil extracellular traps, which together injure the endothelium of dermal, intestinal and glomerular microvessels (2, 4–6). The endothelium is therefore both target and amplifier of injury (7).

A recurring difficulty in the IgAV biomarker literature is specificity. Acute IgAV often features systemic inflammation, including elevated C-reactive protein (CRP), neutrophilia and reactive thrombocytosis, so a raised candidate “endothelial” marker may partly reflect inflammation. Earlier pediatric studies reported increased von Willebrand factor (vWF) in Henoch–Schönlein purpura and associations with inflammatory activity (8, 9). The present study therefore does not seek to establish vWF elevation for the first time. Instead, it evaluates vWF alongside three mechanistically distinct soluble endothelial markers, examines the sensitivity of the vWF result to ELISA plate structure, explores within-patient associations with routine inflammatory indices, and reports the analytical limitations of a parallel endothelial-lineage flow-cytometry readout.

Several soluble analytes are plausible endothelial readouts. vWF is a Weibel–Palade body cargo released during activation; elevated vWF antigen tracks endothelial dysfunction in inflammatory and thromboinflammatory states, including chronic graft-versus-host disease, severe COVID-19, multisystem inflammatory syndrome in children, juvenile dermatomyositis and antineutrophil cytoplasmic antibody (ANCA)-associated vasculitis (10–14). Soluble fms-like tyrosine kinase 1 (sFlt-1) is a decoy receptor for vascular endothelial growth factor (VEGF) that rises in sepsis and renal disease (15, 16); soluble VE-cadherin reflects adherens-junction shedding in overt barrier failure (17, 18); and VEGF drives endothelial proliferation and permeability, with angiopoietin–Tie2 alterations reported in pediatric IgAV (19). These four span complementary endothelial processes: regulated activation/secretion (vWF), VEGF signalling and its decoy (VEGF, sFlt-1), and junctional integrity (VE-cadherin).

Cellular evidence of endothelial damage can in principle be obtained by enumerating circulating endothelial cells (CECs), defined by consensus as CD45^−^CD31^+^CD146^+^ events with confirmed nucleic-acid/viability staining (20, 21). However, CEC enumeration is technically demanding: rare-event detection, gating drift, autofluorescence and inter-laboratory variability all challenge the assay, and standardisation remains incomplete (22). These caveats are especially relevant when, as in routine pediatric practice, fresh samples must be processed on the day of presentation and cannot be batched with concurrently acquired controls. In this study we therefore designate the flow-cytometric readout “endothelial-lineage events” (ELEs) rather than mature CECs.

We performed a cross-sectional pilot study in children with acute IgAV with two aims: (i) to test whether any of four soluble endothelial analytes is altered and to explore whether any observed signal co-varies with systemic inflammatory burden within patients; and (ii) to evaluate, transparently, the performance and limitations of three-colour endothelial-lineage flow cytometry in this clinical setting.

## Materials and methods

### Study design and participants

This single-centre cross-sectional pilot study was conducted at the Pediatric Hospital of the First Affiliated Hospital of Henan University of Chinese Medicine. Twenty consecutive children with newly diagnosed acute IgAV were screened between 2024 and 2025; six were not enrolled because their guardians declined participation, and one was excluded after screening identified abnormal liver function, leaving 13 participants. No formal *a priori* sample-size calculation was performed because this was an exploratory pilot study; the final sample comprised all eligible patients whose guardians provided consent during the recruitment period. Inclusion criteria were: (i) age <18 years; (ii) fulfilment of the EULAR/PRINTO/PReS 2010 (Ankara 2008) classification criteria—mandatory palpable purpura with lower-limb predominance plus ≥1 of diffuse abdominal pain, arthritis/arthralgia, biopsy with predominant IgA deposition, or renal involvement (23); and (iii) sampling during the acute phase before systemic immunosuppressive therapy. All 13 patients met the criteria by purpura plus ≥1 additional feature: seven had joint involvement, ten had gastrointestinal involvement, predominantly abdominal pain and/or hematochezia without severe gastrointestinal complications, and three had renal involvement, all with proteinuria and without renal dysfunction (Table 1). Exclusion criteria were chronic infection, malignancy, primary immunodeficiency, congenital heart disease, clinically significant hepatic dysfunction, prior coagulation disorder and ongoing antiplatelet/anticoagulant therapy.

**Table 1:** Demographic, clinical and laboratory characteristics of the study population.

Characteristic	IgAV (*n* = 13)	HC (*n* = 20)	*P*-value
Age, years—median (IQR)	8.0 (7.0–10.0)	7.5 (4.0–11.0)	0.35
Sex, male/female	7/6	11/9	1.00^a^
Palpable purpura, *n* (%)	13 (100)	—	—
Joint involvement, *n* (%)	7 (54)	—	—
Gastrointestinal involvement, *n* (%)	10 (77)	—	—
Renal involvement, *n* (%)	3 (23)	—	—
Disease duration, days—median (IQR)	9 (7–10)	—	—
Platelets, ×10^9^/L—median (IQR)	394 (307–449)	268 (232–291)	0.001
White blood cells, ×10^9^/L—median (IQR)	7.7 (6.0–14.2)	6.6 (5.9–7.7)	0.15
Neutrophils, %—median (IQR)	66.9 (52.7–77.6)	48.9 (40.6–55.6)	0.004
CRP, mg/L—median (IQR)	5.6 (2.1–8.1)	0.9 (0.6–1.6)	<0.001

Mann–Whitney *U* test unless noted.

a Fisher's exact test. CRP analyses used the recorded numerical values.

Age- and sex-comparable healthy controls (HC) were recruited from the hospital's well-child clinic during the same period. HC were eligible only if they had no acute or recent (within 2 weeks) infection, no allergic disease, no known autoimmune or inflammatory condition, no recent vaccination, and normal routine blood counts and CRP at screening.

### Ethics approval and consent

The study was approved by the Ethics Committee of the First Affiliated Hospital of Henan University of Chinese Medicine (Approval No. 2024HL-437-01). The submitted manuscript represents a cross-sectional baseline analysis of vascular endothelial injury-related markers under an ethics-approved project on vascular endothelial injury-related proteins in pediatric HSP/IgA vasculitis; HSP is the historical term for IgA vasculitis. Written informed consent was obtained from parents or legal guardians; developmentally appropriate children also provided assent. Reporting follows the STROBE guideline for cross-sectional studies (Supplementary File 2).

### Sample collection

Peripheral venous blood was drawn on the day of enrolment using a minimal-trauma technique (21-gauge needle; first 2 mL discarded). Samples for ELISA were collected into EDTA tubes, centrifuged within 30min (1,500 × g, 10 min, 4 °C); plasma was aliquoted and stored at −80 °C until batched analysis. Flow cytometry required a separate fresh K2-EDTA whole-blood specimen processed within 4 h; stored plasma could not be used for this assay. Plasma was available from all 13 IgAV participants and all 20 HC. Fresh whole blood was processed for 12 IgAV participants and 13 HC (HC-08–HC-20). One IgAV sample was insufficient for flow processing; HC-01–HC-07 had plasma ELISA measurements but were not processed for flow cytometry.

### ELISA

Plasma sFlt-1, soluble VE-cadherin, vWF and VEGF were measured by commercial sandwich ELISA according to the manufacturer's instructions. Samples and calibrators were run without replicate wells; optical-density values were used for concentration calculations. The 33 samples were measured across two 96-well plates run approximately one month apart. The two plates were unbalanced with respect to group: plate 1 contained 11 IgAV and 7 HC, and plate 2 contained 2 IgAV and 13 HC. The same kit lot was used across the two runs for each analyte. Each plate carried its own seven-point linear calibration curve (R^2^ 0.991–0.997 across analyte–plate runs), from which concentrations were back-calculated. For vWF and VEGF, samples on plate 1 were diluted 1:5 and reported concentrations were multiplied by five, whereas plate 2 samples were tested neat. Formal dilution-parallelism, recovery and inter-plate bridging experiments were not performed. Because group and plate were partially confounded by this design, the principal comparison (vWF) was subjected to a batch-stratified sensitivity analysis (see Statistical analysis); we do not claim formal inter-plate equivalence. Concentrations are reported in pg/mL (sFlt-1, VEGF) or ng/mL (VE-cadherin, vWF).

### Flow cytometry of endothelial-lineage events

Endothelial-lineage events (ELEs) were quantified as CD45-low CD31^+^CD146^+^ events within a CD45-low/SSC-low gate (P3), using the CD45/CD31/CD146 surface-marker combination reported in prior CEC methods (20, 21). Briefly, 100 µL fresh whole blood was incubated (20 min, 4 °C, dark) with APC anti-CD45 (clone HI30), PE anti-CD31 (clone WM59) and FITC anti-CD146 (clone P1H12) (all BioLegend; Supplementary Table S1). Erythrocytes were lysed, samples washed twice in PBS + 0.5% BSA, and 50,000 total events were acquired on a Beckman Coulter CytoFLEX with daily QC-bead calibration. Manual gating in CytExpert v2.4 comprised FSC-A/SSC-A debris exclusion, doublet exclusion, the CD45-low gate (P3), and the CD31^+^CD146^+^ quadrant within P3. ELEs are reported as a percentage of the P3 gate.

We disclose the following methodological constraints, which bear directly on interpretation. (i) Antibody volumes followed the manufacturer's recommendations, but formal antibody titration, fluorescence-minus-one controls and isotype controls were not performed. No nucleic-acid or viability dye or dedicated platelet-exclusion strategy was used; the three surface markers alone cannot distinguish viable nucleated endothelial cells from membrane fragments, platelet-associated events, aggregates or autofluorescent debris. The events are therefore operationally defined as ELEs rather than confirmed mature CECs. (ii) Cases and controls were acquired non-concurrently because fresh blood was processed on the day of presentation: the 12 IgAV samples were acquired across five sessions and the 13 HC samples in one session. Available records contained one shared raw unstained control for IgAV sessions S1–S4, an archived unstained plot but no corresponding raw FCS file for S5, and one unstained control for the HC session. (iii) Archived FCS files showed the same instrument hardware, fluorescence channels and recorded detector gains, but display scales, gate placement and quadrant thresholds differed across session sets. The CD45-low gate occupied 3.3%–64.9% of events in IgAV versus 0.1%–21.2% in HC. ELE percentages therefore cannot be cleanly separated from session and denominator effects and are treated as exploratory.

### Statistical analysis

Continuous variables are summarised as median (interquartile range, IQR). Distributional form was assessed using the Shapiro–Wilk test and graphical inspection; given the small sample and non-normal or skewed distributions, group differences were tested with the two-sided Mann–Whitney *U* test, reporting the Hodges–Lehmann median difference with 95% confidence interval (CI) and effect size *r* = |*z*|/√*N*. Sex distributions were compared with Fisher's exact test. Because the study was exploratory, both unadjusted *P* values and Benjamini–Hochberg adjusted *q* values are reported for the four soluble endothelial analytes. For vWF, a batch-stratified van Elteren test assessed whether the group difference persisted after accounting for ELISA plate, and the within-plate comparison in the larger (plate 1) batch was reported. Within-IgAV Spearman correlations between vWF and inflammatory markers (CRP, neutrophil proportion, absolute neutrophil count and platelets) were computed using the recorded numerical CRP values, with bias-corrected and accelerated bootstrap 95% CIs (10,000 paired resamples). No observations were excluded as outliers. Two-sided *P* < 0.05 was considered statistically significant. Analyses used Python (SciPy).

## Results

### Cohort characteristics and evidence of systemic inflammation

**Figure 1:**
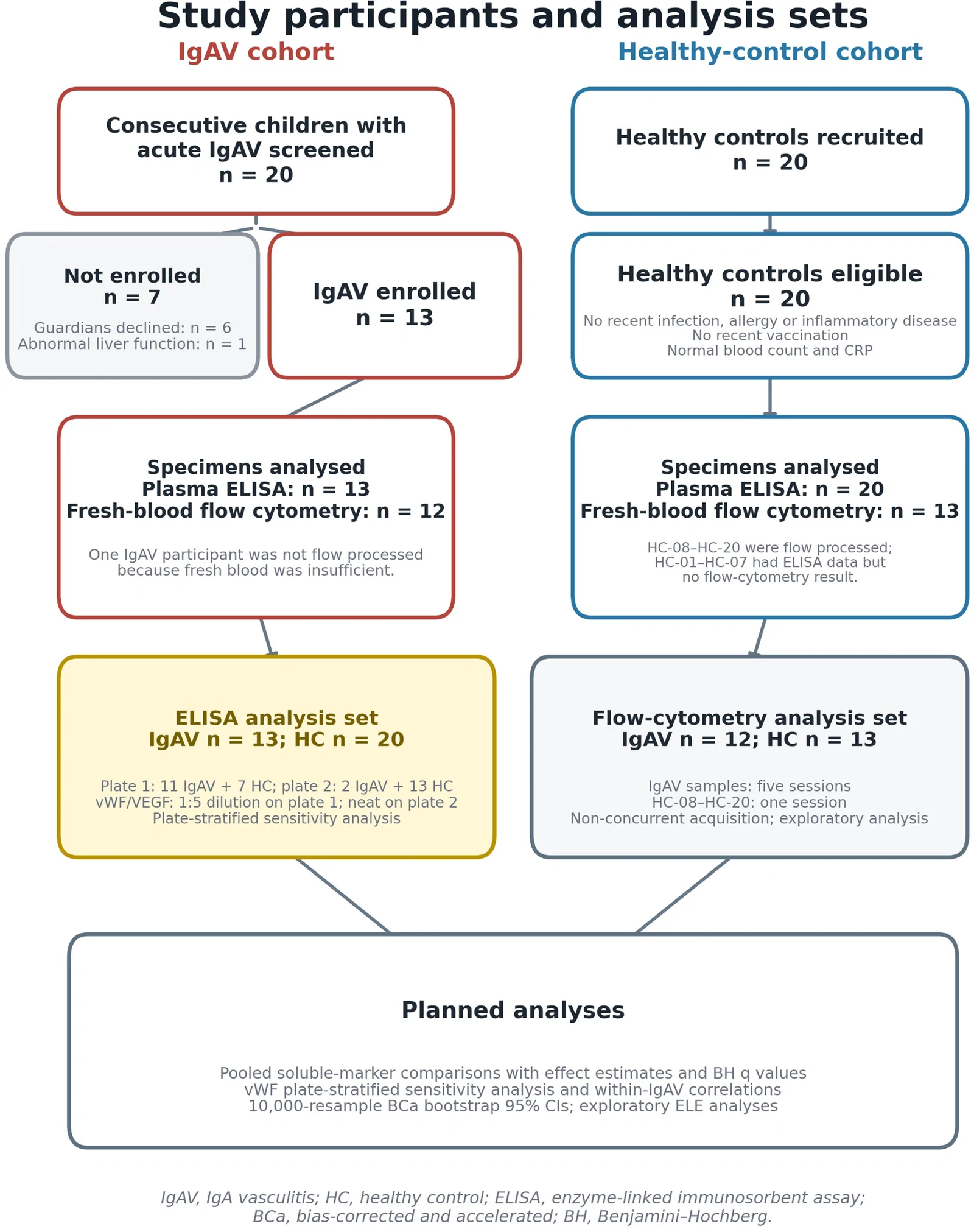
Study flow diagram showing screening, enrolment, specimen availability and assignment of IgAV patients and healthy controls to ELISA and flow-cytometry analyses. All 20 healthy controls contributed plasma; flow cytometry was performed for HC-08–HC-20, whereas HC-01–HC-07 had plasma ELISA measurements but were not processed for flow cytometry.

Of 20 consecutive children with acute IgAV screened, six were not enrolled because their guardians declined participation and one was excluded for abnormal liver function; 13 were enrolled and contributed plasma. Twelve of these 13 contributed fresh blood for flow cytometry (one patient had insufficient fresh blood for flow processing). All 20 HC contributed plasma. Flow cytometry was performed for 13 (HC-08–HC-20); HC-01–HC-07 had plasma ELISA measurements but were not processed for flow cytometry (Figure 1). The groups were comparable in age and sex (Fisher's exact *P* = 1.00 for sex; Table 1). All IgAV patients had palpable purpura; seven had joint involvement, ten had gastrointestinal involvement and three had renal involvement (Table 1). Gastrointestinal disease was predominantly abdominal pain and/or hematochezia without severe complications; the three renal cases had proteinuria without renal dysfunction.

Children with IgAV had higher platelet counts (*P* = 0.001), neutrophil proportions (*P* = 0.004) and CRP than HC; CRP was 5.6 [2.1–8.1] versus 0.9 [0.6–1.6] mg/L (*P* < 0.001) (Table 1). This inflammatory backdrop provides clinical context for the exploratory endothelial-marker analyses below.

### Soluble endothelial markers

**Figure 2:**
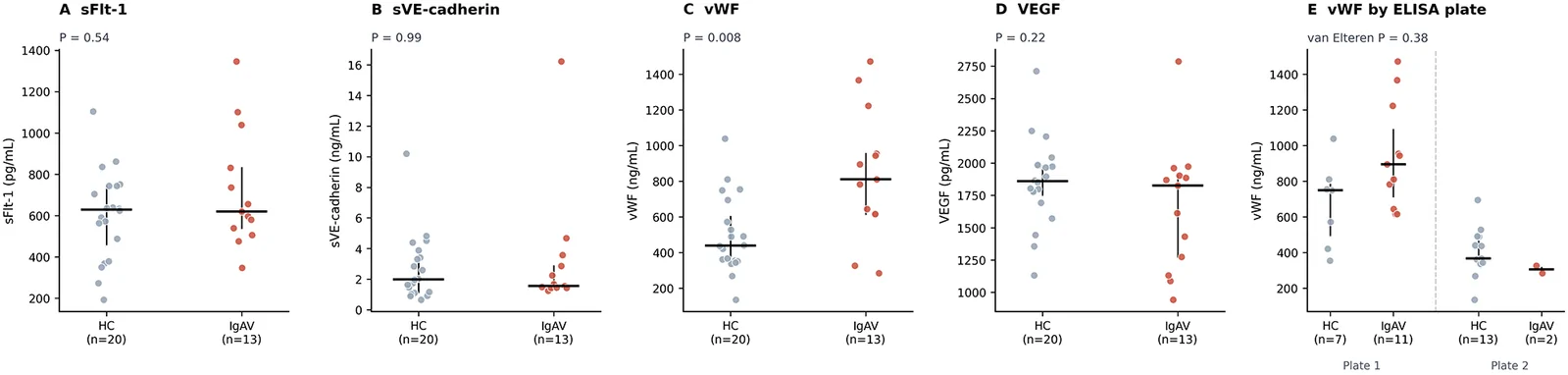
Plasma endothelial markers in IgAV (*n* = 13) and HC (*n* = 20). Dot plots with median and IQR for **(A)** sFlt-1, **(B)** soluble VE-cadherin, **(C)** vWF and **(D)** VEGF; exact *P* values are shown (Mann–Whitney *U* test). **(E)** vWF by ELISA plate, illustrating the group × plate imbalance and the batch-stratified (van Elteren) result.

In the pooled analysis, plasma vWF was higher in IgAV than HC (median 811.1 [IQR 616.7–955.6] vs. 439.9 [354.6–603.0] ng/mL; Hodges–Lehmann difference 335 [95% CI 133–539] ng/mL; *P* = 0.008, *q* = 0.03, *r* = 0.47). No clear group differences were detected for sFlt-1, soluble VE-cadherin or VEGF (all *P* ≥ 0.22 and *q* ≥ 0.43); full estimates are provided in Table 2 and Figures 2A–D.

**Table 2:** Plasma endothelial markers with Hodges–Lehmann differences, Benjamini–Hochberg *q* values and batch-stratified vWF analysis.

Marker	IgAV (*n* = 13)	HC (*n* = 20)	HL diff [95% CI]	*P*	*q*
sFlt-1 (pg/mL)	619.0 (539–831)	628.8 (461–744)	76 [−87, 278]	0.54	0.72
sVE-cadherin (ng/mL)	1.56 (1.45–2.87)	1.99 (1.15–3.54)	0.0 [−1.2, 0.6]	0.99	0.99
vWF (ng/mL)	811.1 (617–956)	439.9 (355–603)	335 [133, 539]	0.008	0.03
VEGF (pg/mL)	1,825 (1,275–1,904)	1,860 (1,755–1,982)	−153 [−530, 75]	0.22	0.43
vWF—batch sensitivity					
Plate 1 (11 vs. 7 HC)	894	750	—	0.077	—
Plate 2 (2 vs. 13 HC)	306	367	—	0.17	—
van Elteren (stratified)	—	—	—	0.38	—

Values are median (IQR) unless noted. HL diff = Hodges–Lehmann median difference (IgAV − HC). *q* = Benjamini–Hochberg adjusted *P* across the four analytes. The pooled vWF difference does not persist after batch stratification (van Elteren *P* = 0.38).

### vWF: batch sensitivity

Because the two ELISA plates were unbalanced by group, we tested whether the vWF difference persisted after batch stratification. It did not: the van Elteren batch-stratified test gave *P* = 0.38, and within the larger single-plate batch (11 IgAV vs. 7 HC) the difference was a non-significant trend (median 894 vs. 750 ng/mL; *P* = 0.077) (Figure 2E). The two IgAV patients measured on plate 2 had low vWF values (286 and 327 ng/mL), comparable to plate-2 HC. We therefore interpret the pooled vWF elevation as hypothesis-generating and requiring batch-balanced confirmation. The within-patient analyses below are also exploratory because they include only 13 participants and retain the two-plate measurement structure.

### Within-patient association between vWF and inflammation

**Figure 3:**
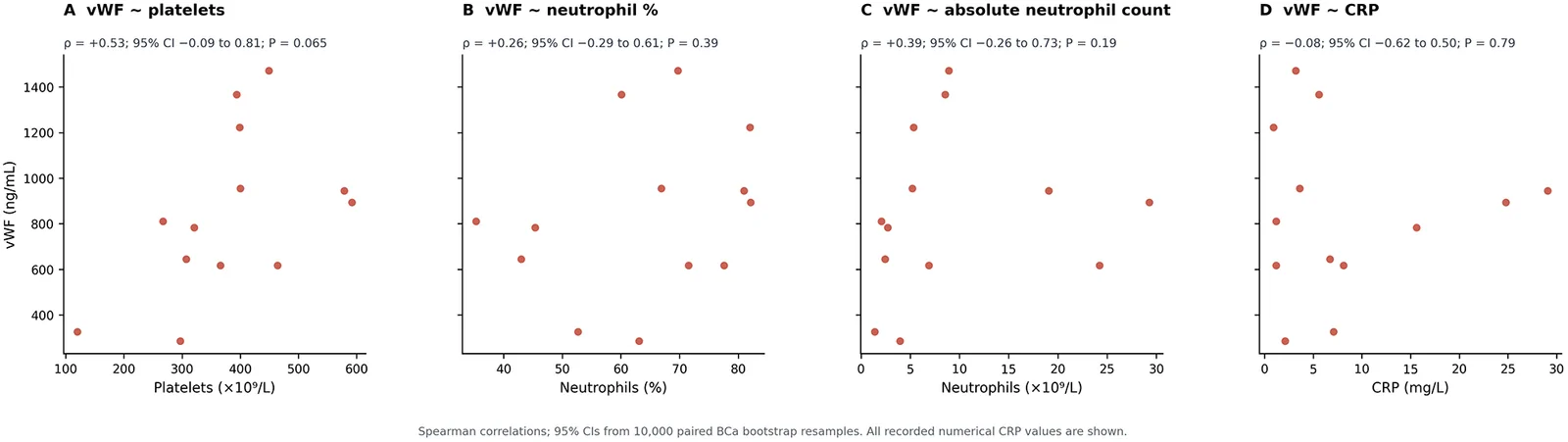
Within-IgAV correlations (*n* = 13) between vWF and markers of systemic inflammation: **(A)** platelets, **(B)** neutrophil proportion, **(C)** absolute neutrophil count, and **(D)** CRP. Spearman *ρ* with bias-corrected and accelerated bootstrap 95% CI is shown.

Within the IgAV group, no clear monotonic association was detected between vWF and CRP (Spearman *ρ* = −0.08, 95% CI −0.62 to 0.50; *P* = 0.79), neutrophil proportion (*ρ* = 0.26, 95% CI −0.29 to 0.61; *P* = 0.39), absolute neutrophil count (*ρ* = 0.39, 95% CI −0.26 to 0.73; *P* = 0.19), or platelet count (*ρ* = 0.53, 95% CI −0.09 to 0.81; *P* = 0.065) (Figure 3). All bootstrap CIs were wide and spanned zero. These underpowered analyses are compatible with no association but also with clinically meaningful associations and therefore cannot establish that vWF is independent of systemic inflammation.

### Endothelial-lineage events: exploratory and batch-sensitive

**Figure 4:**
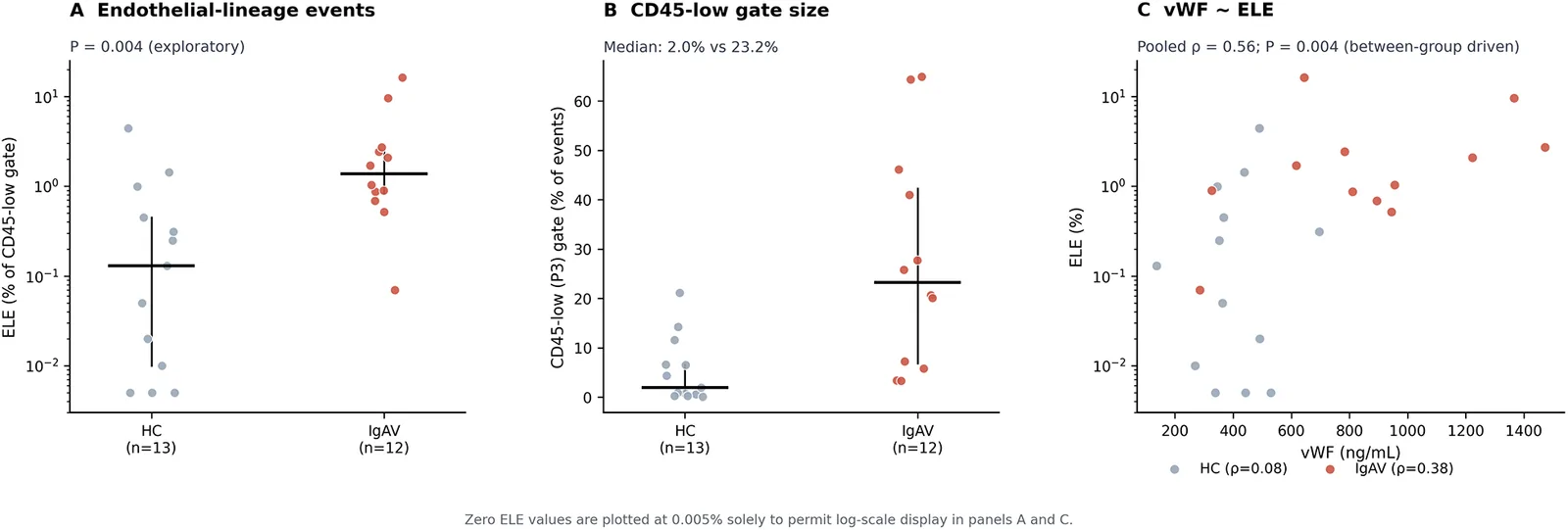
Endothelial-lineage events (ELEs). **(A)** ELE% (log scale) in IgAV (*n* = 12) versus HC (*n* = 13). **(B)** CD45-low (P3) gate size by group, illustrating its wide and group-divergent range. **(C)** vWF–ELE correlation, pooled and within group. Exact *P* values are shown. Zero ELE values are plotted at 0.005% solely to permit log-scale display in panels **(A)** and **(C)**.

ELEs were nominally higher in IgAV than HC (1.37% [0.83–2.50] vs. 0.13% [0.01–0.45]; Hodges–Lehmann difference 0.91% [95% CI 0.47–2.27]; *P* = 0.004, *r* = 0.58) (Table 3; Figure 4A). However, this statistical separation does not overcome the lack of analytical comparability between groups. IgAV ELE values were heterogeneous (range 0.07%–16.37%), and six of 12 values (≤1.1%) overlapped the HC range. The CD45-low (P3) gate on which the percentage depends varied between samples from 0.1% to 64.9% and was systematically larger in IgAV (median 23.2%) than HC (median 2.0%), so the denominators were not comparable (Figure 4B). Cases and controls were acquired in separate sessions, gate placement and quadrant thresholds were not standardised, and formal titration, FMO/isotype controls, viability/nucleic-acid staining and platelet exclusion were absent (Methods). ELE% did not correlate clearly with inflammatory markers within IgAV (all |*ρ*|<0.30). The observed group difference is retained as a descriptive, exploratory result but is not interpreted as definitive biological evidence of increased circulating endothelial cells.

**Table 3:** Endothelial-lineage events (ELEs) and CD45-low gate in IgAV versus HC.

Parameter	IgAV (*n* = 12)	HC (*n* = 13)	*P*
ELE, % of CD45-low gate—median (IQR)	1.37 (0.83–2.50)	0.13 (0.01–0.45)	0.004
ELE range, %	0.07–16.37	0.00–4.44	—
IgAV values overlapping HC range (≤1.1%)	6/12	—	—
CD45-low (P3) gate, %—median (range)	23.2 (3.3–64.9)	2.0 (0.1–21.2)	—

Hodges–Lehmann difference 0.91% [95% CI 0.47–2.27]; *r* = 0.58. Reported as exploratory: percentages depend on a CD45-low gate that varied 0.1%–64.9% and differed by group; cases and controls were acquired in separate sessions without a prospectively standardised gate/control strategy.

### Association between vWF and ELEs

Across the 25 subjects with paired data, vWF and ELE% were positively correlated (Spearman *ρ* = 0.56, 95% CI 0.25–0.76; *P* = 0.004), but within-group correlations were not significant (HC *ρ* = 0.08, 95% CI −0.50 to 0.64; IgAV *ρ* = 0.38, 95% CI −0.32 to 0.87) (Figure 4C). The pooled correlation may reflect between-group structure rather than within-group covariation; because both readouts are batch-sensitive, it is presented descriptively and not assigned mechanistic significance.

## Discussion

In a consecutively enrolled pilot cohort of children with acute IgAV, pooled plasma vWF was higher than in HC, but the case–control contrast was sensitive to ELISA plate imbalance. No clear within-patient associations were detected between vWF and routine inflammatory indices, although these analyses were too small to establish biological independence. No clear group differences were detected in sFlt-1, soluble VE-cadherin or VEGF. The endothelial-lineage flow-cytometric readout produced a nominal group difference that was strongly affected by gating and acquisition-session structure. We discuss each finding in turn and outline practical methodological implications.

### vWF and its relationship to inflammation

Increased vWF in pediatric Henoch–Schönlein purpura was reported previously, including an association with inflammatory activity (8, 9). Accordingly, the contribution of the present pilot study is not the first demonstration of vWF elevation. Its added value lies in comparing vWF with three mechanistically distinct endothelial analytes, showing how the pooled vWF contrast changes after explicit plate stratification, exploring within-patient correlations using individual-level data, and pairing the soluble-marker work with a transparent assessment of a technically challenging cellular readout. The lack of statistically significant correlations with CRP or neutrophil indices should not be read as evidence that vWF is independent of inflammation: with *n* = 13 and wide bootstrap intervals, the data remain compatible with no association as well as potentially meaningful positive associations. Batch-balanced and adequately powered studies are required to determine whether vWF provides information beyond routine inflammatory markers.

### Why the other soluble markers were unchanged

The three analytes without clear case–control differences are mechanistically distinct from vWF. Soluble VE-cadherin reflects proteolytic shedding, whereas endothelial activation can also cause VE-cadherin internalisation and junctional redistribution without necessarily increasing circulating soluble VE-cadherin; its absence from plasma therefore does not exclude endothelial perturbation. sFlt-1 may rise chiefly in more generalised microvascular dysfunction (15, 16), and circulating VEGF is sensitive to disease context and pre-analytical platelet release. The predominantly skin-, joint- and gastrointestinal-involved phenotype of this small cohort may not have produced detectable changes in these axes, but limited power and assay variability also remain plausible explanations. These negative results should therefore be interpreted as an absence of detected differences rather than proof of unchanged biology.

### Methodological lessons from the flow-cytometric data

The flow-cytometric data should be interpreted as an exploratory endothelial-lineage signal rather than definitive enumeration of mature circulating endothelial cells. The observed pattern is useful primarily for defining technical requirements for future pediatric IgAV studies: prospective concurrent case–control acquisition; instrument performance tracking; pre-specified and blinded gate templates; formal antibody titration; FMO and appropriate control strategies; viability and nucleic-acid discrimination; exclusion of platelet-associated events and aggregates; and absolute rare-event counting (20–22). A three-colour CD45/CD31/CD146 surface panel without these safeguards cannot confirm viable nucleated endothelial cells. Reporting events as a percentage of a CD45-low denominator that varied from 0.1% to 64.9% further undermines comparability. Thus, the nominal group difference remains a hypothesis-generating observation, while the present records clarify the quality-control requirements for a definitive study.

### Limitations

First, no formal *a priori* sample-size calculation was performed, and the sample was small (13 IgAV; 12 with flow cytometry); estimates and correlation CIs are therefore imprecise. Second, the cross-sectional design precludes inference about temporal dynamics or causality. Third, ELISA group allocation was unbalanced across plates, vWF/VEGF dilution differed between plates, and no formal dilution-parallelism, recovery or inter-plate bridging experiment was performed. The stratified analysis shows that the pooled vWF difference does not persist under plate control. Fourth, ABO blood group, an important determinant of vWF concentration, was not collected. Fifth, clinical heterogeneity could not be analysed meaningfully: ten patients had gastrointestinal involvement without severe complications, and the three renal cases had proteinuria without renal dysfunction. Their individual vWF values (783.33, 944.44 and 894.44 ng/mL) fell within the overall patient range and showed no obvious separation, but formal subgroup inference is not warranted. Sixth, flow samples were acquired non-concurrently and lacked formal titration, FMO/isotype controls, viability/nucleic-acid staining, platelet exclusion and an absolute-count strategy; the CD45-low denominator also varied widely. Finally, this was a single-centre study, and endothelial-cell enumeration varies between laboratories (22).

## Conclusions

In this small cross-sectional pilot study of children with acute IgAV, pooled plasma vWF was higher than in healthy controls, but the contrast was sensitive to ELISA plate imbalance and should be considered hypothesis-generating. No clear within-patient associations were detected between vWF and CRP or neutrophil indices; the wide confidence intervals preclude a conclusion that vWF is independent of systemic inflammation. No clear group differences were detected in sFlt-1, soluble VE-cadherin or VEGF. Three-colour endothelial-lineage flow cytometry produced a nominal group difference, but gate-size and acquisition-session variability prevent definitive biological interpretation. These observations support prospective, batch-balanced validation of vWF, including antigen and functional activity, together with longitudinal assessment against disease activity, renal involvement and treatment response, and rigorously standardised endothelial-cell enumeration.

## Data Availability

The original contributions presented in the study are included in the article/Supplementary Material, further inquiries can be directed to the corresponding author.

## Supplementary material

Supplementary Table S1 Antibody clones, fluorochromes, vendors and working-volume information used for flow cytometry.

Supplementary Table S2 Per-subject raw data: vWF, sFlt-1, soluble VE-cadherin, VEGF, ELE%, CD45-low gate size, ELISA plate and flow acquisition session.

Supplementary Table S3 Per-plate ELISA standard-curve parameters and group composition per plate.

Supplementary Table S4 Flow-cytometry acquisition sessions, available unstained controls and recorded instrument/session features.

Supplementary File 2 STROBE checklist for cross-sectional studies.
